# Evaluating risk factor assumptions: a simulation-based approach

**DOI:** 10.1186/1472-6947-11-55

**Published:** 2011-09-07

**Authors:** Carolyn M Rutter, Diana L Miglioretti, James E Savarino

**Affiliations:** 1 Biostatistics Unit, Group Health Research Institute, Seattle, WA, USA; 2 Department of Biostatistics, University of Washington, Seattle, WA, USA

**Keywords:** microsimulation, colorectal cancer, comparative effectiveness, screening

## Abstract

**Background:**

Microsimulation models are an important tool for estimating the comparative effectiveness of interventions through prediction of individual-level disease outcomes for a hypothetical population. To estimate the effectiveness of interventions targeted toward high risk groups, the mechanism by which risk factors influence the natural history of disease must be specified. We propose a method for evaluating these risk factor assumptions as part of model-building.

**Methods:**

We used simulation studies to examine the impact of risk factor assumptions on the relative rate (RR) of colorectal cancer (CRC) incidence and mortality for a cohort with a risk factor compared to a cohort without the risk factor using an extension of the CRC-SPIN model for colorectal cancer. We also compared the impact of changing age at initiation of screening colonoscopy for different risk mechanisms.

**Results:**

Across CRC-specific risk factor mechanisms, the RR of CRC incidence and mortality decreased (towards one) with increasing age. The rate of change in RRs across age groups depended on both the risk factor mechanism and the strength of the risk factor effect. Increased non-CRC mortality attenuated the effect of CRC-specific risk factors on the RR of CRC when both were present. For each risk factor mechanism, earlier initiation of screening resulted in more life years gained, though the magnitude of life years gained varied across risk mechanisms.

**Conclusions:**

Simulation studies can provide insight into both the effect of risk factor assumptions on model predictions and the type of data needed to calibrate risk factor models.

## 1.0 Background

Microsimulation models describe events and outcomes at the person-level and provide policy-relevant information by predicting the population-level impact of different interventions [1]. For example, microsimulation models can be used to predict trends in disease incidence and mortality under alternative health policy scenarios [2,3] or to compare the clinical effectiveness and cost-effectiveness of treatments [4]. Several risk factors play a role in colorectal cancer, including both non-modifiable factors such as age, sex, race, and family history and modifiable risk factors related to lifestyle including diet, activity level, and medication use [5,6]. Microsimulation models can be used to examine the impact of patient-centered interventions that are focused on risk reduction and interventions targeted to high risk individuals, such as more intensive colorectal cancer (CRC) screening for individuals at higher risk, with initiation at earlier ages and/or shorter screening intervals [7]. Models that include risk factors must make assumptions about how risk factors affect specific disease processes and the magnitude of these effects [7-9]. For example, two microsimulation models for CRC include detailed risk factor components including factors related to increased risk (body mass index, smoking status, red meat consumption) and factors related reduced risk (physical activity, fruit and vegetable consumption, multivitamin use, aspirin use, hormone replacement therapy). One model allows these factors to affect only adenoma occurrence [2,10], the other allows risk factors to affect both adenoma occurrence and progression to preclinical CRC [11]. These risk factor models are complex, requiring description of changes in modifiable risk factors over time, yet basic work to inform risk factor modeling is lacking and little is known about the relationship between different assumptions and conclusions about the effectiveness of interventions. This is especially important because data available to distinguish between different risk mechanisms is sparse.

Race is an example of a relatively simple risk factor that we wish to include in natural history models for colorectal cancer [7,11]. Race is an imperfect proxy measure of genetic and lifestyle factors [12] associated with increased risk of CRC. There are demonstrated racial disparities in CRC outcomes. African Americans have higher CRC incidence and mortality than non-Hispanic whites, particularly at younger ages, and are more likely to be diagnosed with late stage disease [13]. The overall CRC incidence rate ratio for African American versus non-Hispanic white groups is 1.21 among men and 1.26 among women [14]. In addition, African Americans tend to be diagnosed with later stage CRC than whites [13,15,16] and at a younger age [17]. There is scant direct information about the reasons for these disparities [18]. Some studies suggest that African American and non-Hispanic white groups have similar adenoma prevalence [18,19]. Colonoscopy studies have found that African Americans are more likely to have large adenomas than non-Hispanic whites [20]. Therefore, race could plausibly be modeled as a risk factor that is related to adenoma incidence, growth, or progression to colorectal cancer. We describe a method for exploring the impact of these risk factor assumptions on model results, which provides insight into the data required to build a model that includes a risk factors.

## 2.0 Methods

We consider the impact of risk factors in the context of the Colorectal Cancer Simulated Population model for Incidence and Natural history (CRC-SPIN) [21]. CRC-SPIN simulates the natural history of colorectal cancer arising from adenomas (Table 1). For each simulated individual, the CRC-SPIN model generates a time of birth and a time of non-CRC (other-cause) death. Within this lifetime, adenoma occurrence is simulated using a non-homogeneous Poisson process with risk that systematically varies as a function of gender and age. Each initiated adenoma is stochastically assigned a location in the large intestine and a time to reach 10 mm (which may exceed the individual's lifetime). Adenoma growth depends on adenoma location (colon or rectum) and is modeled using a continuous process with an asymmetric growth curve that specifies exponential growth early that slows as adenomas reach a maximum of 50 mm. The probability of adenoma transition to preclinical cancer is a function of adenoma size, adenoma location (colon or rectum), gender, and age at adenoma initiation. The time of transition from preclinical cancer to clinical detection due to the onset of symptoms in the absence of screening, sojourn time, depends on the location of the preclinical cancer (colon or rectum). Once a cancer becomes clinically detectable, the size, stage at clinical detection, and survival are simulated based on SEER data. Details about the model structure and calibration are provided elsewhere, [21,22] below we focus on incorporation of risk factors into this basic model.

**Table 1:** Basic structure of the CRC-SPIN Model

Model component	
**Adenoma incidence: non-homogeneous Poisson process**	
Log-risk for the *i*th individual at time *t*, ℓn(Ψ*_i_*(*t*)) =	
$\alpha_{0i}+\alpha_{1}sex_{i}+\overset{4}{\underset{k=1}{\sum }}\delta \left(A_{k}<age_{i}\left(t\right)\leq A_{k+1}\right)\left\{age_{i}\left(t\right)\alpha_{2k}+\overset{k}{\underset{j=2}{\sum }}A_{j}\left(\alpha_{2,j-1}-\alpha_{2j}\right)\right\}$	
• Baseline log-risk, α_0i_, is Normally distributed, mean Λ, standard deviation σ • δ(.) is an indicator function with δ(*x*) = 1 when the condition *x *is true and δ(*x*) = 0 otherwise • *age_i_*(*t*) is the *i*th individual's age at time *t* • A_1 _= 20, A_2 _= 50, A_3 _= 60, A_4 _= 70, A_5_=∞ (effectively 100 years old)	
Adenoma location probabilities: cecum: 0.08; ascending colon: 0.23; transverse colon: 24; descending colon: 0.12; sigmoid colon: 0.24; rectum: 0.09.	
**Adenoma growth: Janoshek growth curve**	

*_dij_*(*t*) = *d*_∞ _- (*d*_∞ _- *d*_0_) exp (-*λ_ij_t*) • *d*_ij_(*t*) is the maximum diameter of the *j*th adenoma in the *i*th individual at time *t *after initiation. • *d*_0 _= 1 mm, minimal detectable adenoma size • *d*_∞ _= 50 mm, maximum adenoma size • time to reach 10 mm: -In((*d*_∞ _- 10)/(*d*_∞ _- *d*_0_))/*λ*.	
**Time to reach 10 mm: type 2 extreme value distribution**	

Adenomas in the colon: distribution parameterized by β_1c _and β_2c_	
Adenomas in the rectum: distribution parameterized by β_1r _and β_2r_	
**Transition to preclinical cancer: normal cumulative distribution**	

Probability of transition, male colon	Ф({In (*γ_1 cm _size*) + *γ_2 cm _*(*a*-50)}/*γ*_3_
Probability of transition, male rectum	Ф({In (*γ_1 rm _size*) + *γ_2 rm _*(*a*-50)}/*γ*_3_
Probability of transition, female colon	Ф({In (*γ_1 cf _size*) + *γ_2 cf _*(*a*-50)}/*γ*_3_
Probability of transition, female rectum	Ф({In (*γ_1 rf _size*) + *γ_2 rf _*(*a*-50)}/*γ*_3_
Where Ф(.) is the standard Normal cumulative distribution function, *size *is adenoma size in mm, and *a *is age at adenoma initiation.	
**Sojourn time: lognormal distribution**	

Preclinical colon cancer, lognormal with mean μ_c_, standard deviation τ_c_μ_c_	
Preclinical rectal cancer, lognormal with mean μ_r_, standard deviation τ_r_μ_r_	

### 2.1 Risk factors

For simplicity, we consider a single binary risk factor indicated by *x_i_*(*t*) for the *i*th individual at time *t *where *x_i_*(*t*) = 1 if the risk factor is present at time *t *and *x_i_*(*t*) = 0 otherwise. We allow this risk factor to affect adenoma incidence, adenoma growth, transition to clinically detectable cancer, sojourn time, and other cause mortality. We do not allow risk factors to affect CRC survival given stage at detection.

#### Adenoma risk

Let *Ψ_I _*(*t*) be the *i*th individual's risk of developing an adenoma at time *t *in the absence of any risk factor. This model has a proportional hazards structure, so that adenoma risk can be written as exp[*δ_1 _x _I _*(*t*)]*Ψ_I _*(*t*). The number of adenomas an individual develops by time *t *has a Poisson distribution, and the expected number of adenomas for an individual with a fixed risk factor is exp[δ_1_] times greater than the expected number for an individual of the same age and gender without the risk factor.

#### Adenoma growth

Let *d*(*t*) be adenoma size at time *t *in the absence of any risk factor. We incorporate risk factors into the adenoma growth model by assuming that the effect of risk factors is to either expand or contract the time scale, *t*, so that given risk factor information, adenoma size is a function of *t*' (*x_i _*(*t*)) = exp (*δ*_2_*x_i_*(*t*)) *t*. Under this model, adenomas in an individual with a fixed risk factor reach a given size (e.g., 10 mm) in exp(-δ_2_) the time it takes adenomas to reach the same size in similar individuals without the risk factor.

#### Transition from adenoma to cancer

The probability of transition from adenoma to cancer is a function of gender, the location of the adenoma, the age at adenoma initiation, and the size of the adenoma with the probability of transition to cancer increasing with increasing adenoma size. We incorporate risk factors into the adenoma transition model by assuming that the effect of risk factors is to either expand or contract the size scale. Given risk factor information, the probability of transition is a function of $s_{ij}^{\prime }\left(x_{i}\left(t\right)\right)=\overset{s}{\underset{0}{\int }}\exp\left(\delta_{3}x_{i}\left(u\right)\right)du$. Let P(*s *| *age, location, gender*) be the probability that an adenoma of size *s *in an individual without the risk factor transitions to cancer given their *gender*, the *location *of the adenoma and the *age *at adenoma initiation. Under this model, if a person with the same characteristics also had the risk factor, then the probability that an adenoma of size *s *transitions to cancer is equal to exp(δ_3_)P(*s *| *age, location, gender*). For an individual with a fixed adenoma transition risk factor, the increase in risk is Ф((ln(γ_1_exp(δ_3_)*s*) + γ_2_(*a*-50))/γ_3_) - Ф((ln(γ_1_s) + γ_2_(*a*-50))/γ_3_) where *a *is age at adenoma initiation in years, γ_1 _and γ_2 _are location- and gender-specific transition parameters (see Table 1), and Ф(.) is the standard Normal cumulative distribution function.

#### Sojourn time (time to clinical detection)

In contrast to adenoma growth and transition to preclinical cancer, which may take a decade or more to occur, transition from preclinical cancer to clinically detectable cancer is believed to occur within about 5 years [23-25]. We assume that only those risk factors present at the time of transition to preclinical cancer affect sojourn time, and assume that they have a multiplicative effect on sojourn time. Let *ST_ij _*be sojourn time for the *i*th individual's *j*th preclinical cancer in the absence of risk factor information. If *x*_i _indicates a risk factor present at the time of transition to preclinical cancer, then sojourn time is given by *ST'_ij_*(*x_i_*) = exp[δ_4 _*x_i_*]*ST _ij_*.

#### Other-cause mortality

Risk factors that increase risk for CRC such as high body mass index and smoking [10] may also increase risk of death from other causes. The CRC-SPIN model stochastically assigns age at other-cause (non-CRC) death based on data from the National Center for Health Statistics with survival that depends on gender and birth cohort [26]. Let *a _i _*be the age at other-cause death for the *i*th individual in the absence of risk factor information. For a dichotomous risk factor, we allow the change in age at other cause death to be proportional to the time the risk factor is present, so that $a_{i}^{\prime }\left(x_{i}\left(t\right)\right)=\overset{a}{\underset{0}{\int }}\exp\left(\delta_{5}x_{i}\left(u\right)\right)du$. Under this model, the age at other-cause death given a fixed risk factor *x_i _*is *a'_j_*(*x_i_*) = exp[δ_5 _*x_i_*]*a _j_*.

### 2.2 Simulation studies

We conducted two simulation studies to explore the effect of risk factor mechanisms on CRC incidence and mortality and screening effectiveness assuming a single dichotomous risk factor that is present or absent for an individuals' entire life (e.g., race).

The first simulation study examined the effect of different risk factor mechanisms on predicted CRC outcomes. We simulated 10 scenarios: no risk factors present, presence of one of four CRC-specific risk factor mechanisms, increased other-cause mortality, and increased other-cause mortality in combination with one of the four CRC-risk factor mechanisms. The four CRC-specific risk factor mechanisms were: increased adenoma risk, faster adenoma growth, more likely and earlier progression to cancer, and shorter sojourn time. To simulate increased adenoma risk we specified a 10%, 25%, 50%, or 100% increase in adenoma incidence (corresponding to exp(δ_1_) = 1.10, 1.25, 1.50, and 2.0, respectively). To simulate faster adenoma growth we specified a 10%, 20%, 30%, or 50% reduction in the time to reach 10 mm (corresponding to exp(-δ_2_) = 0.9, 0.8, 0.7, and 0.5, respectively). To simulate more likely and earlier progression to cancer we specified transition rates comparable to an adenoma that is 5%, 10%, 20%, or 30% larger (corresponding to exp(δ_3_) = 1.05, 1.10, 1.20, and 1.30, respectively). To simulate shorter sojourn time we specified a 10%, 20%, 50%, or 75% reduction in the time from preclinical cancer to clinical detection (corresponding to exp(δ_4_) = 0.9, 0.8, 0.5, and 0.25, respectively). To simulate increased other-cause mortality we specified a 5%, 10%, 15%, or 20% reduction in survival due to death from other causes (corresponding to exp(δ_5_) = 0.95, 0.9, 0.85, and 0.8, respectively). Simulations that examined the combined effect of CRC-specific risk factors and increased other-cause mortality focused on a 10% reduction in survival due to death from other causes.

For each scenario and each level of risk factor within scenario we simulated a cohort of 10 million individuals aged 45 years for each of 1,000 simulated draws from the posterior distribution of our model parameters. We aged individuals in the cohort for 35 years, though some died before reaching age 80. For each of our 1,000 draws, we estimated the mean for each outcome and 95% prediction intervals based on upper and lower 2.5*th *percentiles. Prediction intervals reflect variability in predictions resulting from estimated parameters and variability due to estimation of sample statistics. Simulated sample sizes were chosen to be large to minimize sampling variability.

We conducted a simulation study to evaluate the effects of risk factors on intermediate disease processes related to the risk factor mechanisms: average time from adenoma onset to preclinical CRC (preclinical dwell time), the proportion of adenomas that transition to preclinical cancer, the proportion of preclinical cancers that transition to clinically detectable cancer, and the average time of transition from preclinical cancer to clinically detectable cancer (sojourn time). Next, we estimated the relative rate (RR) of CRC incidence and mortality and 95% prediction intervals for cohorts with each of the 9 risk factor scenarios compared to a cohort with no risk factors. We calculated relative rates for both the 30-year period from age 45 to 79 years (the 'overall' RR) and for three age groups (45-54, 55-64, 65-79). This is analogous to a hypothetical observational study that enrolls individuals at age 45, with 35 years of follow-up.

Our second simulation study examined the effect of different risk factor mechanisms on predicted efficacy of screening colonoscopy, focusing on a subset of risk factor mechanisms and strengths that resulted in similar increased rates of CRC. We simulated four risk factor scenarios: a no risk factor scenario and three scenarios with increased CRC risk (100% increase in adenoma incidence, 30% faster adenoma growth, and transition probabilities as if adenomas were 30% larger). We simulated two colonoscopy screening regimens. The first regimen reflects current guidelines with screening beginning at age 50 and subsequent screening every 10 years. The second screening regimen assumes earlier initiation with screening beginning at age 45 and subsequent screening every 10 years. For both regimens we assumed 100% compliance, with the screening regimen, including surveillance colonoscopy at 3 or 5 years based on the number and size of adenomas detected at screening, consistent with current guidelines. If screening detected three or more adenomas or at least one adenoma that is 10 mm or larger, then the individual returns for colonoscopy in three years. If screening detects one or two adenomas that are each less than 10 mm large, then the individual returns for colonoscopy in five years. If no adenomas are found, then the individual continues routine screening and returns for colonoscopy in ten years. For each risk factor scenario and screening regimen we examined the RR of CRC incidence and mortality along with 95% prediction intervals for those with and without screening.

When simulating colonoscopy, we assumed that adenoma miss rates decreased with size. Based on observed miss rates [27-29], we modeled the probability of missing an adenoma or cancer given its size as P(miss|size = *s *and *s*< 20 mm) = 0.34-0.035*s *+ 0.0009*s*^2^, where *s *is adenoma diameter in mm. All adenoma and cancers 20 mm and larger were assumed to be detected by colonoscopy. The associated miss rates for adenomas and cancers that are 1 mm, 5 mm, 10 mm, and 15 mm in size were 31%, 19%, 8%, and 2%. We also assumed incomplete reach of the scope, with 90% of simulated colonoscopy exams complete to the cecum,[30] 5% complete to the ascending colon, 3% complete to the transverse colon, and 2% complete to the descending colon.

For each risk factor scenario and level, and each of 1,000 model parameter values, we simulated a cohort of 10 million 45 year old individuals. For each risk factor cohort, we calculated the mortality reduction (MR) and life years gained (LYG) per 1000 individuals attributable to screening by comparing screened to unscreened cohorts up the each individual's 80^th ^birthday. MR represents the proportional reduction in colorectal cancer deaths and LYG representing the difference in survival following the age at screening initiation. Finally, we compared the predicted impact of different screening regimens across risk factor cohorts. We estimated posterior means, based on the average predictions across 1,000 simulated parameter values; 95% prediction intervals, based on 2.5*th *and 97.5*th *percentiles of predictions across 1,000 simulated parameter values; and the posterior probability of differential effectiveness of different screening regimens for different risk cohorts, based on the percentage of times screening was more effective in one cohort compared to another across 1,0000 simulated parameter values [31].

## 3.0 Results

Simulated intermediate (and not directly observable) outcomes were consistent with risk factor assumptions, with CRC-specific risk factors affecting only their associated mechanisms (data not shown). Increased adenoma incidence resulted in greater adenoma prevalence and decreased sojourn time resulted in a higher probability of transition from preclinical to clinical CRC and shorter mean sojourn time. Increased adenoma growth and increased probability of transition from adenoma to preclinical cancer had similar effects with both resulting in a higher probability of transition to preclinical cancer and shorter mean preclinical dwell time. The similarity of these effects is consistent with the structure of the CRC-SPIN model, which assumes that the probability of transition to preclinical CRC increases with adenoma size. Increased risk of other-cause death resulted in a lower probability of transition to preclinical CRC, slightly shorter preclinical dwell time, a slightly lower probability of transition from preclinical to clinical CRC, and a slight shortening of sojourn time. This occurs because individuals with longer preclinical dwell and sojourn times are more likely to die before transition to the next disease state.

For a given risk factor mechanism and strength of effect, the RR of CRC incidence with versus without the risk factor was nearly identical to the RR of CRC morality with versus without the risk factor. For example, when adenomas grew 30% faster, the mean RR of CRC incidence versus CRC mortality was: 1.84 versus 1.85 for ages 45-79; 2.34 versus 2.37 for ages 45-54; 2.00 versus 2.03 for ages 55-64; and 1.72 versus 1.73 for ages 65-79. Across the range of scenarios explored, differences in RRs for CRC mortality and incidence ranged from -0.04 to 0.14, with 77% of the scenarios showing a 0.01 or smaller difference in RRs. Given this similarity, the remainder of our paper focuses exclusively on CRC mortality.

The RR of CRC mortality associated with presence of a CRC-specific risk factor decreased towards one with increasing age, and the rate of change across age groups depended on the risk factor mechanism (Table 2). For example, there was a greater change in RR across age groups for faster adenoma growth than for increased adenoma incidence, even when the risk factors were set to levels that produced similar overall increases in CRC mortality. The rate of change increased with the strength of the risk factor. A 100% increase in adenoma incidence resulted in a 1.89 RR of CRC mortality from age 45 to 79, and the RR ranged from 1.98 for ages 45-54 down to 1.86 for ages 65-79. Similarly, 30% faster adenoma growth resulted in a 1.85 RR of CRC mortality from age 45 to 79, but for this mechanism the RR ranged from 2.37 for ages 45-54 down to 1.73 for ages 65-79. The effects of faster adenoma growth and transition to preclinical cancer at smaller adenoma sizes on the RR were similar. Reduced sojourn time had little impact on the RR of CRC incidence or mortality.

**Table 2:** Estimated impact of a hypothetical fixed risk factor on CRC mortality, with 95% prediction intervals

	CRC mortality			
	
Risk factor	45-79 years old	45-54 years old	55-64 years old	65-79 years old
**Baseline risk, δ_1_=δ_2_=δ_3_=δ_4_=δ_5 _= 0**				

CRC mortality rate	175 (150,212)	13 (10,20)	43 (35,56)	121 (105,1142)

	**Rate ratios relative to a cohort without the risk factor**			

**Increased adenoma incidence**				

10% more adenomas	1.09 (1.08,1.10)	1.10 (1.07,1.12)	1.10 (1.08,1.11)	1.09 (1.08,1.10)
25% more adenomas	1.23 (1.21,1.25)	1.25 (1.22,1.27)	1.24 (1.22,1.26)	1.23 (1.20,1.25)
50% more adenomas	1.45 (1.41,1.49)	1.49 (1.46,1.52)	1.48 (1.44,1.51)	1.45 (1.40,1.49)
100% more adenomas	1.89 (1.79,1.96)	1.98 (1.92,2.02)	1.95 (1.87,2.00)	1.86 (1.75,1.96)

**Faster adenoma growth**				

10% faster	1.22 (1.18,1.26)	1.31 (1.23,1.39)	1.25 (1.19,1.30)	1.20 (1.16,1.23)
20% faster	1.49 (1.39,1.59)	1.75 (1.53,1.93)	1.59 (1.46,1.71)	1.44 (1.35,1.52)
30% faster	1.85 (1.67,2.02)	2.37 (1.95,2.75)	2.03 (1.77,2.26)	1.73 (1.58,1.89)
50% faster	2.88 (2.43,3.34)	4.57 (3.30,5.81)	3.39 (2.75,4.04)	2.54 (2.17,2.93)

**Preclinical transition at smaller sizes**				

As if 5% larger	1.13 (1.11,1.15)	1.18 (1.13,1.22)	1.15 (1.12,1.18)	1.12 (1.10,1.14)
As if 10% larger	1.26 (1.22,1.30)	1.37 (1.28,1.45)	1.30 (1.24,1.36)	1.24 (1.19,1.28)
As if 20% larger	1.53 (1.43,1.62)	1.80 (1.59,1.98)	1.64 (1.50,1.75)	1.48 (1.39,1.56)
As if 30% larger	1.81 (1.66,1.96)	2.28 (1.92,2.59)	1.98 (1.76,2.18)	1.71 (1.57,1.85)

**Shorter sojourn time**				

10% reduction	1.01 (1.01,1.03)	1.02 (1.00,1.05)	1.02 (1.00,1.03)	1.01 (1.00,1.03)
20% reduction	1.03 (1.01,1.05)	1.05 (1.01,1.09)	1.04 (1.02,1.06)	1.03 (1.01,1.05)
50% reduction	1.08 (1.04,1.12)	1.13 (1.06,1.22)	1.09 (1.05,1.15)	1.07 (1.04,1.12)
75% reduction	1.12 (1.07,1.19)	1.20 (1.11,1.34)	1.14 (1.08,1.24)	1.10 (1.06,1.18)

**Other-cause survival**				

5% reduction	0.95 (0.94,0.95)	1.00 (0.97,1.02)	1.00 (0.98,1.01)	0.98 (0.96,0.99)
10% reduction	0.87 (0.85,0.88)	0.99 (0.97,1.02)	0.99 (0.98,1.01)	0.94 (0.93,0.96)
15% reduction	0.76 (0.73,0.78)	0.99 (0.97,1.01)	0.98 (0.97,1.00)	0.89 (0.87,0.90)
20% reduction	0.63 (0.60,0.66)	0.98 (0.96,1.01)	0.97 (0.95,0.99)	0.81 (0.79,0.84)

The table shows the CRC death rates per 10,000 life years in the cohort without risk factors, and relative rates comparing CRC death rates cohorts with risk factors to cohorts without risk factors. 95% prediction intervals are shown in parenthesis.

Increased other-cause mortality reduced the risk of CRC mortality (RR < 1, Table 2), a result of competing risks. The reduction in risk increased with both increasing other-cause mortality and increasing age. Collapsing across age groups results in greater estimated risk reduction (across the 45-79 year age range) than age-specific RRs, demonstrating Simpson's paradox [32,33]. We observed similar patterns when we modeled co-occurrence of CRC-specific risk factors and increased mortality. In the presence of increased risk for other-cause mortality, age specific RRs associated with colorectal cancer were slightly attenuated, with greater degrees of observed attenuation for RR across the whole 45-79 year old age range (data not shown). For example, a 10% reduction in other-cause survival reduced the RR associated with a 100% increase in adenoma incidence from 1.89 down to 1.64 (95% prediction interval (PI) (1.57,1.71)) overall and from 1.86 down to 1.76 (95% PI (1.66,1.85)) in the 65-79 year old age group, with no evidence of attenuation of the RR in the 45-54 and 55-64 year old age groups.

We explored the potential for different risk factor mechanisms to impact predicted screening effectiveness across three types of risk factors associated with a similar increased risk of CRC mortality (compared to the no risk factor group: 1.89 RR for increased adenoma risk, 1.85 RR for faster adenoma growth, and 1.81 RR for transition at smaller sizes). There were small differences in the predicted CRC mortality reduction (Table 3) and life years gained (Table 4) across risk factor mechanisms. The ordering of these differences was consistent with small differences in the overall relative risk of CRC mortality. In all cases, earlier initiation of screening resulted in both a greater mortality reduction and more life years gained per 1000 individuals screened (LYG). Across risk groups (including the no increased risk group), initiating screening at age 45 resulted in a 5 percentage point increase in mortality reduction compared to initiation at age 50.

**Table 3:** CRC mortality reduction by risk factor cohort and screening regimen, relative to no screening

	Age at screening initiation (years)	
	**50**	**45**

**Risk factors:**		

None	0.90 (0.87,0.92)	0.95 (0.92,0.96)
100% more adenomas	0.89 (0.86,0.92)	0.96 (0.94,0.97)
30% faster adenoma growth	0.86 (0.83,0.88)	0.92 (0.89,0.94)
Preclinical transition sizes as if 30% larger	0.86 (0.83,0.88)	0.92 (0.89,0.94)

Predictions are shown with 95% prediction intervals.

**Table 4:** Life years gained per 1000 individuals screened compared to no screening by risk factor cohort and screening regimen: Mean predictions are shown with 95% prediction intervals

	Age at initiation (years)		
	**50**	**45**	Difference*

**Risk Factors:**			

None	128 (108, 155)	146 (122, 181)	18 (14, 26)
100% more adenomas	248 (210, 298)	281 (236, 346)	33 (25, 48)
30% faster adenoma growth	236 (198, 272)	276 (230, 322)	41 (21, 51)
Preclinical transition sizes as if 30% larger	232 (198, 267)	271 (229, 317)	39 (31, 50)

* Estimated difference in life years gained per 1000 individuals with screening initiated at age 45 versus initiation at age 50 years.

We also examined the differential LYG for screening initiation at 45 versus 50 years. There were larger increases in LYG due to screening in risk factor cohorts compared to the no risk cohort, ranging from 15 more years per 1000 individuals screened (95% PI (11,22)) for cohorts with more adenomas to 22 more years per 1000 individuals (95% PI (16,28)) for cohorts with faster growing adenomas, though overall the impact of earlier screening on LYG was very small. In spite of having a slightly lower overall risk for CRC mortality, we found larger differences in LYG for cohorts with faster growing adenomas and adenoma transition at smaller sizes than cohorts with more adenomas: Cohorts with faster adenomas had a mean of 8 more LYG per 1000 individuals screened (95% CR (0, 13) compared to cohorts without the risk factor, posterior probability *p *= 0.023); Cohorts with adenomas that transition to cancer at smaller sizes had a mean of 6 more LYG per 1000 individuals screened (95% CR (0, 11), posterior probability *p *= 0.027.

## 4.0 Discussion

Risk factors provide clinical information that can potentially be used to target screening and treatment [7]. Microsimulation models provide a method for evaluating the comparative effectiveness of such targeted interventions, but this requires that modelers make specific assumptions about how risk factors influence the natural history of disease.

We demonstrated the use of simulation studies to explore risk factor assumptions. Simulating different mechanisms by which a hypothetical risk factor affects the natural history of CRC allowed us to explore the impact of risk factor assumptions within the CRC-SPIN model, and was an important step toward building a comprehensive risk factor model. We found that age-specific CRC incidence and mortality and the rate of change in disease incidence across age groups are important sources of information about risk factor mechanisms. For the CRC-SPIN model, the rate of change in CRC incidence and mortality across age groups was smaller for risk factors associated with disease initiation (adenoma risk), with larger rates of change for risk factors associated with disease progress (adenoma growth and transition to preclinical cancer). This indicates that calibration to age-specific outcomes is critical for risk factor models. Age-specific information is especially important when modeling risk factors that affect both CRC risk and other-cause survival.

While the predicted mortality reduction and life years gained attributed to screening did not vary much across risk factor assumptions, there were small differences in the predicted increase in life years gained resulting from a shift to screening initiation at age 45 rather than a later initiation at age 50 years. The gains attributed to earlier screening were small, especially in the group without risk factors.

These findings should be considered in the context of our analyses, which focused on the effect of risk factor assumptions on model predictions. We did not estimate costs associated with screening, nor costs per life year gained. Cost considerations add another layer of assumptions and may affect policy decisions related to risk-stratified screening. Our simulations assumed perfect adherence to screening recommendations, but adherence may vary across risk groups. For example, African Americans are at increase risk of CRC relative to non-Hispanic whites, but are also less likely to undergo screening for CRC [34,35]. In addition, we only allowed adenoma size and reach of scope to affect the accuracy of colonoscopy. Other factors, including inadequate preparation, shorter scope withdrawal time, and adenoma type (e.g., flat adenomas) and proximal location may reduce the accuracy of colonoscopy and hence reduce its effectiveness, and presence of these factors may vary across risk groups. The occurrence of de-novo cancer, without a precursor adenoma, could also impact simulated effectiveness. While the CRC-SPIN model allows adenomas to transition to preclinical cancer at any size, the model does not explicitly simulate de-novo cancers. Estimating the impact of these clinical and behavioral factors on colonoscopy effectiveness is an active area of research.

Our study is limited by its focus on a single microsimulation model, and exploration of a single risk factor that was present for an individual's entire lifetime. This focus was motivated by our desire to explore possible mechanisms for increased risk of colorectal cancer incidence in African Americans, who have approximately 1.2 times the risk of CRC relative to non-Hispanic whites, near the low end of the range we explored in our simulations. While this type of non-modifiable risk factor is of interest, modifiable risk factors such as smoking and body mass index are also important and incorporating these modifiable risks into models requires additional assumptions about how changes in risk factors over an individual's lifetime influence the natural history of disease. For example, the time lag between changes in risk factor status and observed changes in risk may be related to the assumed risk factor mechanism. Simulations that inform model choices may be even more important when considering these more complex risk factor structures. We restricted our attention to a risk factor that affects one component of the process leading to diagnosis with colorectal cancer and possibly other-cause (non-colorectal cancer) survival. Our approach to modeling other-cause survival was also fairly simplistic. We suggested this approach primarily for purposes of exploring the possible interaction between risk factors that affect both other-cause survival and CRC natural history processes. We did not allow risk factors to also affect survival after diagnosis with colorectal cancer, an assumption that is consistent with current modeling approaches [2,11], but likely drove the similarity of our findings for colorectal cancer incidence and mortality. Our estimates of screening benefit would change if a risk factor was allowed affect both CRC incidence and survival from CRC. For example, there is some evidence that African Americans have poorer stage-specific CRC survival than non-Hispanic whites [36].

## 5.0 Conclusions

Our study demonstrates the importance of careful evaluation of risk factor assumptions, and the ability to use simulation studies to carry out exploratory analyses. In addition to providing information about the effect of risk factor assumptions on model results, simulation studies can provide insight into the type of data needed to calibrate risk factor models. Gaining clarity about the data needed to calibrate risk factor models is an important first step toward collaborations between modelers and clinician researchers to develop data-driven risk factor models. Finally, these results demonstrate the importance of including a clear description of assumed risk factor mechanisms in studies using microsimulation models, along with sensitivity analyses showing how these assumptions influence results.

## Competing interests

The authors declare that they have no competing interests.

## Authors' contributions

CMR developed the microsimulation model used to demonstrate risk factor assumptions, designed the simulation study used to assess risk factor effects, and drafted the manuscript. DLM assisted in development of the CRC-SPIN model, design of the simulation study, and manuscript development. JES was the programming architect for the CRC-SPIN algorithm, developed and implemented all of the programs used to generate and summarize results. All authors read and approved the final manuscript.

## Pre-publication history

The pre-publication history for this paper can be accessed here:

http://www.biomedcentral.com/1472-6947/11/55/prepub
